# Investigating the evolution and features of regeneration using cnidarians

**DOI:** 10.1093/icb/icaf006

**Published:** 2025-03-17

**Authors:** Aide Macias-Muñoz

**Affiliations:** Department of Ecology and Evolutionary Biology, University of California, Santa Cruz, CA 95064, USA

## Abstract

The ability to regenerate can greatly vary between animal groups and cell types. Some of the outstanding questions in the field of regeneration include: (1) How has regeneration evolved? and (2) What features underlie differences in regeneration potential between animals? Whether regeneration evolved once and diversified or if it evolved multiple times independently by co-opting similar pathways remains unknown. Current research seeks to identify conserved cellular and molecular features that allow for regeneration. However, comparisons between distantly related regenerating animals have revealed a large amount of diversity. In this perspective, I review discussions on the mechanisms, cell types, and genes underlying regeneration. I propose using Cnidaria as a group in which to investigate the evolution of regeneration. As the sister group to Bilateria with notable regenerative capacity, studies in Cnidaria offer insights into the evolutionary history and conservation of regenerative mechanisms. I then highlight how genome-wide studies, single-cell genomics, multi-omics, and gene editing can be used to identify cell types and unknown features of regeneration. Applying these approaches across organisms will give insight into the cell and molecular features that allow for regeneration competency and may be used to alter an organism’s regeneration potential.

## Introduction

Regeneration is the ability to regrow missing body parts. In animals, the extent of regeneration can be variable for different cell types, tissues, and especially between species. As regeneration can require growth and patterning similar to development, many studies focus on a candidate gene approach to identify conserved developmental genes and pathways involved in regeneration. Early fundamental studies using *in situ* hybridization, chemical inhibitions, and even gene knockdowns identified genes essential for regeneration (Endo et al. 1997; Hobmayer et al. 2000). Yet, whether there is a defined set of competency factors—mechanisms, cell types, genes, and genetic pathways—shared across species remains unknown. Of special interest is identifying the cellular and molecular underpinnings of regeneration and characterizing conserved features of regenerating animals. Still unresolved and important to this topic is determining whether regeneration has evolved once and diversified or whether it has evolved more than once and undergone convergence (Brockes and Kumar 2008; Bely and Nyberg 2010; Tiozzo and Copley 2015; Maden 2018; Elchaninov et al. 2021; Srivastava 2021). Understanding the features of regeneration competency and its evolution will provide insight into mechanisms that may also advance the field of regenerative medicine (Alvarado and Tsonis 2006).

**Fig. 1:**
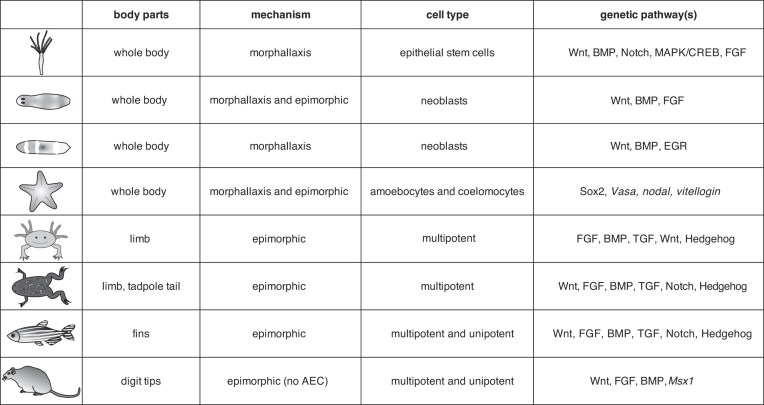
Features of regenerating animals. Summary of tissues, mechanisms, cell types, and genetic pathways of regeneration for some organisms typically used in regeneration studies: *Hydra*, planaria, *Hofstenia*, echinoderms, axolotl, *Xenopus*, zebrafish, and mice. Looking across organisms reveals that there may be diversity in the factors that underlie the ability to regenerate. The use of different genetic pathways for regeneration may suggest multiple independent origins (convergent evolution) of the trait.

In determining whether the ability to regenerate is an ancestral trait, we would assume homology in the features of regeneration, such as the mechanisms, cell types, and genetic pathways involved. A traditional separation of regenerating animals is the division of vertebrates, some of which are capable of limb regeneration, and invertebrates, some of which are capable of whole-body regeneration. This traditional separation is a remnant from early studies on *Hydra* and planarians to a pivot toward using vertebrate model organisms to understand the principles of regeneration (see review Elchaninov et al. 2021). This separation may also be due to whole-body regeneration being possible in some invertebrate animals but absent in all extant vertebrate species. The division of invertebrate and vertebrate regenerating organisms supported a hypothesis that regeneration competency decreases as organisms become more complex in structure and have more derived traits (Zhao et al. 2016). Hypotheses as to the differences of regeneration competency include differences in the complexity of tissues, immune systems, life cycles, and development (Aztekin and Storer 2022). For example, invertebrate models for regeneration have stem cells, and vertebrate animals capable of regeneration have cells that can undergo dedifferentiation and transdifferentiation; these mechanisms are limited in animals that are not able to regenerate (Tanaka and Reddien 2011). Likewise, animals with more complex immune systems seem to have more difficulty undergoing regeneration (Zhao et al. 2016; Elchaninov et al. 2021; Aztekin and Storer 2022). Complex immune systems are those that include scarring and inflammation and possess specialized immune cells with signaling molecules that coordinate the immune response (Zhao et al. 2016). While some comparative studies have identified signaling pathways that are similar between model organisms for regeneration (Somorjai et al. 2012; Darnet et al. 2019), studies in regenerating organisms across taxa have revealed a large amount of diversity (Fig. 1) (Endo et al. 1997; Chera et al. 2011; Lehoczky et al. 2011; Galliot 2012; Sanders and Kent 2014; Srivastava et al. 2014; Oulhen et al. 2016; Dolan et al. 2018; Aztekin et al. 2019; Gehrke et al. 2019; Ivankovic et al. 2019; Bölük et al. 2022; Zheng et al. 2022). It should be noted that this comparison is only a subset of regenerating animals, model organisms that are typically used for comparative cellular and genetic studies. A comprehensive comparison may yield additional diversity. Even comparisons of related organisms reveal additional levels of variation. As an example, *Hydra* belong to the phylum Cnidaria, which are diploblastic radial animals, sister to Bilateria. *Hydra* are capable of whole-body regeneration (Trembley 1744). Yet, cnidarians vary in life history strategies, and not all have the same regenerative ability (discussed below). Similarly, while some planarians are capable of full body regeneration, others may have lost the ability to regenerate (Ivankovic et al. 2019). In echinoderms, the tissues that can regenerate, the timing, cells, and the mechanisms used to regenerate vary between different groups (Mashanov et al. 2008; Hernroth et al. 2010; Reinardy et al. 2015; Oulhen et al. 2016; Khadra et al. 2018).

Regeneration has traditionally been divided into two mechanisms: morphallaxis and epimorphic. Morphallaxis is a process that does not require cell proliferation but rather rearrangement of existing cells. Epimorphic regeneration, on the other hand, requires the formation of a group of undifferentiated cells at the injury site called a blastema. Model organisms for regeneration are traditionally associated with these mechanisms, such as *Hydra* with morphallaxis and amphibians with epimorphosis. Cells in the *Hydra* polyp are constantly undergoing mitosis and being shed at the extremities. When*Hydra* are bisected, epithelial cells close the wound within 6 h, and a new head with tentacles is fully formed within 72 h (Bode 2003). Early studies found no difference in the mitotic rate of intact *Hydra* and a *Hydra* undergoing head regeneration, so it was assumed that head regrowth occurred through existing cell movement (morphallaxis) (Park et al. 1970). However, it was later found that *Hydra* bisected in the mid-gastric region have an area of cell proliferation that is similar to a blastema (Chera et al. 2009). Blastema formation, commonly associated with amphibians, is a feature that defines epimorphic regeneration. After the loss of a limb or tail, amphibians exhibit wound closure by epidermal cells, these apical-epithelial-cap cells signal to a group of undifferentiated cells (termed the blastema). Progenitor cells in the blastema undergo proliferation and differentiation (Brockes 1997; Aztekin and Storer 2022). Similar to *Hydra*, blastema-like structures have been identified in some planarians, echinoderms, and lancelets (Somorjai et al. 2012; Khadra et al. 2018; Ivankovic et al. 2019). Since both mechanisms can be found in the same animal group and even in the same organisms, it was proposed that these concepts be redefined (Agata et al. 2007). It has also been suggested that the epimorphic process evolved from morphallaxis (Bely and Nyberg 2010; Elchaninov et al. 2021). Authors hypothesize this evolutionary history of the processes due to their underlying similarities, but epimorphic being a “bilaterian innovation” (Bely and Nyberg 2010; Elchaninov et al. 2021). However, as mentioned above, blastema-like structure, typical of epimorphic regeneration, has been discovered in *Hydra*, a non-bilaterian. This highlights the importance of correctly defining the mechanisms of regeneration and identifying their homology to unravel whether these mechanisms evolved once or multiple times.

Another important feature for regenerating animals is the types of cells that can be used to regrow missing body parts. Some animals, such as *Hydra* and planarians, can use pluripotent stem cells, while others, such as vertebrates, make use of a mix of multipotent and/or unipotent cells. These multipotent or unipotent progenitor cells can arise from dedifferentiation, transdifferentiation, or migration. The comparative cellular underpinnings of regenerating organisms have been reviewed in the literature and highlight many unknowns (Tanaka and Reddien 2011; Storer and Miller 2020; Srivastava 2021). As an example, the contribution of amoebocytes and coelomocytes as pluripotent cells in different echinoderms is still unresolved (Khadra et al. 2018). Similarly, heterogenous multipotent and unipotent cells are still being identified in regenerating complex structures such as limbs in amphibians and digit tips in mice (Storer and Miller 2020; Aztekin and Storer 2022). Moreover, the organizing cells for regenerating tissues and their signaling components have also yet to be characterized. Characterization of organizing cells and cells used for regeneration will give further insight into the mechanisms of regeneration and potential cell homologies (Arendt et al. 2016; Shafer 2019; Srivastava 2021).

A third component of interest in determining the evolution of regeneration is the genetic and signaling pathways underlying this complex process. The field of evolutionary development (EvoDevo) highlighted similarities in the molecular underpinnings of development across organisms (Carroll 2008). EvoDevo theory posits that complex trait evolution has a deep homology and novel traits arise by making use of established genes and gene networks (Carroll 2008; Monteiro and Podlaha 2009; Shubin et al. 2009). As regeneration requires tissue patterning, similar to development, many researchers have focused on conserved developmental pathways to investigate their role in regeneration. Such studies have found a role in regeneration for pathways including Wnt, MAPK/JNK, FGF, TGF, and BMP signaling (Hobmayer et al. 2000; Srivastava et al. 2014; Scimone et al. 2016; Aztekin et al. 2019; Darnet et al. 2019). However, while some of the genes in these pathways may result in a loss of regeneration when inhibited or knocked down, the complete gene regulatory networks and their conservation across organisms remain unknown (Srivastava 2021). An alternative model to regeneration being homologous is that regeneration arose convergently and makes use of similar but non-orthologous genes through co-option of a different member of the gene family (paralog switching), co-option of different genes, or use of different gene regulatory networks. To determine the evolutionary history of regeneration, we need to incorporate a phylogenetic framework in investigating the homology of cellular and molecular mechanisms underlying this complex process (see Srivastava 2021). The availability of genomic tools in non-model organisms and advances in transgenics can now help us decipher some of the missing links or additional diversity in the molecular underpinnings of regeneration.

Here, I highlight how comparative approaches and new technologies can help resolve some of the unknown factors of regeneration. Firstly, I propose an expansion of comparative studies into non-model organisms and suggest a broadening of cnidarian species used to further investigate the features of regeneration. Next, I discuss insights gained by genome-wide and multi-omics approaches and the limitations of current studies using high-throughput sequencing. Lastly, I emphasize how new tools and approaches can be used to identify unknown features of regeneration. One such advancement in the field is the rise of single-cell sequencing that can now be used to identify cell types and trajectories associated with regeneration. Another advancement is the application of CRISPR to generate transgenics in non-model organisms, which can be used to functionally validate candidate genes and pathways. We can now make broad comparisons between closely related species and across taxa to identify components of regeneration that are conserved or co-opted, thus giving insight into the evolution of this complex trait.

## Cnidaria as a model to investigate regeneration

Cnidaria, the phylum that *Hydra* belong to, includes corals, sea anemones, and jellyfish. As a sister group to Bilateria (the group that encompasses all bilateral animals), studies in cnidarians can give insight into the evolution of genomes, gene networks, and gene functions. In addition to their phylogenetic placement, cnidarians are of interest in the scientific community due to their venomous nature (Klompen et al. 2021), eye complexity (Picciani et al. 2018), and regenerating capabilities (Galliot and Schmid 2002). A rise in popularity of cnidarian research has resulted in new resources for comparative and genetic studies, such as genome assemblies (Putnam et al. 2007; Chapman et al. 2010; Gold et al. 2019; Leclère et al. 2019; Ohdera et al. 2019; Cazet et al. 2023; Zimmermann et al. 2023; Schnitzler et al. 2024) and single-cell atlases (Sebé-Pedrós et al. 2018; Siebert et al. 2019; Chari et al. 2021) (see Table 1). These resources along with the unique biology of cnidarians present an opportunity to investigate the features and evolutionary history of regeneration. While some species such as *Nematostella, Hydra, Hydractinia, Clytia*, and *Turritopsis* have quite a few tools to build from, many species outside of Hydrozoan remain underrepresented and should be developed for comparative approaches.

**Table 1:** Publicly available cnidarian resources.

Group	Species	Genome (NCBI RefSeq)	Number of scaffolds	Single-cell atlas	Transgenic methods
Anthozoa	*Nematostella vectensis*	Chromosome-level: GCA_033964005.1	29	Yes	https://doi.org/10.1242/dev.204387
	*Acropora digitifera*	Genome assembly: GCF_000222465.1	2420	No	N/A
	*Exaiptasia diaphana*	Genome assembly: GCF_001417965.1	4312	No	N/A
	*Anthopleura elegantissima*	Genome assembly: GCA_042767785.1	4216	No	N/A
	*Edwardsiella lineata*	none	-	No	N/A
Hydrozoa	*Hydra vulgaris*	Chromosome level (AEP): GCF_038396675.1	15	Yes	https://doi.org/10.1007/978-1-0716-2172-1_34
	*Hydra oligactis*	Genome assembly: GCA_024195425.1	16,310	No	N/A
	*Hydractinia symbiolongicarpus*	Chromosome level: GCF_029227915.1	78	Yes	https://doi.org/10.1186/s12864-018-5032-z
	*Clytia hemisphaerica*	Genome assembly: GCF_902728285.1	1396	Yes	https://doi.org/10.1038/s41598-018-30188-0
	*Turritopsis rubra*	Genome assembly: GCA_039566895.2	326	Yes	https://doi.org/10.1038/s41467-024-49848-z
Scyphozoa	*Aurelia aurita*	Genome assembly: GCA_004194415.1	2709	No	N/A
	*Cassiopea xamachana*	Genome assembly: GCA_964235115.1	561	No	N/A
	*Chrysaora fuscescens*	Genome assembly: GCA_009936425.2	295,560	No	N/A
	*Rhizostoma pulmo*	none	-	No	N/A
Cubozoa	*Tripedalia cystophora*	none	-	No	N/A
	*Alatina alata*	Genome assembly: GCA_008930755.2	2,005,718	No	N/A
	*Chironex flecker*	none	-	No	N/A
	*Carybdea rastonii*	none	-	No	N/A
Staurozoa	*Haliclystus sanjuanensis*	none	-	No	N/A
	*Lucernaria quadricornis*	none	-	No	N/A
	*Craterolophus convolvulus*	none	-	No	N/A
	*Calvadosia cruxmelitensis*	Genome assembly: GCA_900245855.1	50,999	No	N/A

**Fig. 2:**
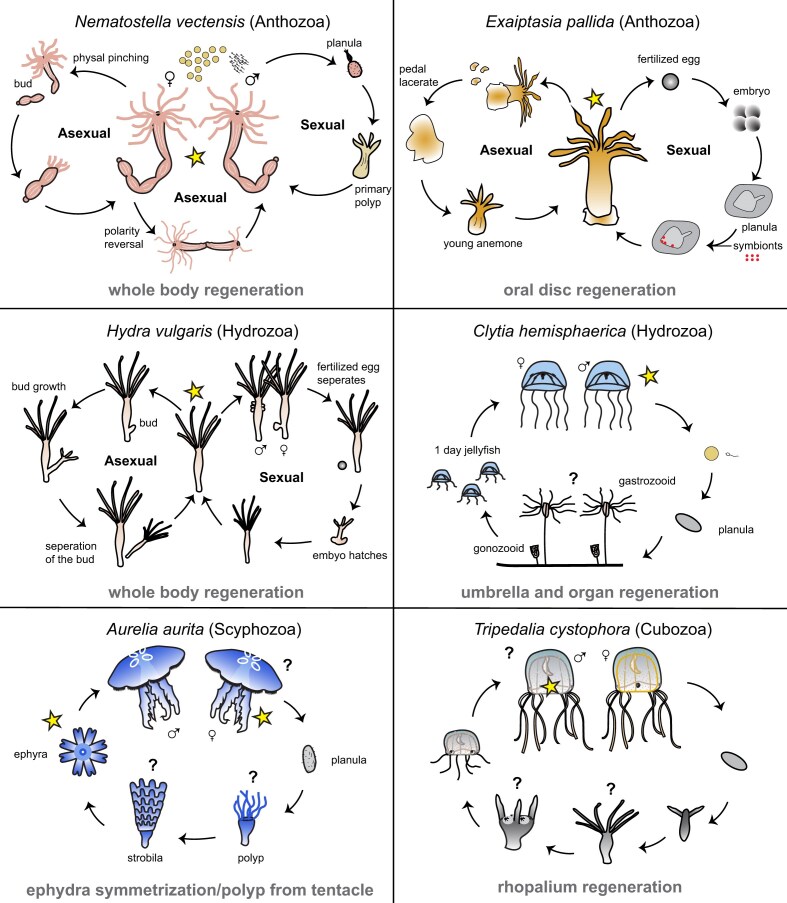
Cnidarian life cycles. *Nematostella* can reproduce sexually, asexually by physal pinching and polarity reversal, and are capable of full body regeneration, drawing adapted from Kelava 2014. *Exaipatasia* can reproduce sexually, asexually by pedal lacerates, and can regenerate their oral disc, drawing adapted from the Guse lab website. *Hydra* can reproduce sexually, asexually by budding, and are capable of full body regeneration, drawing adapted from Schaible et al. 2017. *Clytia* have external fertilization that proceeds by a polyp colony to produce medusae and are capable of umbrella and organ regeneration, drawing adapted from Wikimedia Commons by Munro. *Aurelia* has external fertilization that proceeds by a strobila structure. They are capable of regeneration of polyp from tentacles and of ephyra symmetrization, drawing adapted from Matveev et al. 2012. *Tripedalia* polyps reproduce asexually, and each polyp undergoes metamorphosis into an individual medusa. They are capable of fully regenerating their sensory structure called a rhopalium, drawing adapted from Gurska and Gram 2014. Stars indicate known life stages where regeneration has been investigated. Question marks denote life stages where regeneration is possible, but the extent of regenerative capacity is yet unresolved.

It remains unknown whether life cycle complexities affect regeneration competency. Cnidarians have diverse life cycles, which may influence their ability and their method of regeneration (Collins 2002). To our knowledge, all cnidarians are capable of asexual reproduction, which may facilitate the ability to regenerate during some developmental stages. Gaps in knowledge include whether stem cells are present across cnidarians (Gold and Jacobs 2013) and the extent to which medusae can regenerate. A comparison of cnidarian species commonly used in the field of biology reveals differences in their life cycles (Fig. 2). *Nematostella* and *Exaiptaisia* are anthozoan species that have external fertilization that proceeds to a planula larva. However, while *Exaiptaisia* can produce copies of itself from pedal lacerates that grow into a young anemone (Presnell et al. 2022), *Nematostella* is capable of asexual fission by physal pinching and polarity reversal (Reitzel et al. 2007). Within the group Hydrozoa, two commonly studied species include *Hydra* and *Clytia*. Under favorable conditions, *Hydra* reproduces asexually by budding, but under stressful conditions hermaphroditic *Hydra* undergoes gamete production. In this case, eggs are fertilized within the female, and an embryo is released that hatches into a young *Hydra* (Galliot and Schmid 2002; Steele et al. 2019). On the contrary, *Clytia* has a medusa adult stage, external fertilization, a planula stage, and a polyp colony (Leclère et al. 2019). In addition to Hydrozoa, the group Medusozoa has two other classes: Cubozoa (box jellyfish) and Scyphozoa (true jellyfish). The box jellyfish *Tripedalia* has a life cycle that includes internal fertilization, and each polyp undergoes metamorphosis to become an adult medusa (Werner et al. 1971). Unlike *Tripedalia*, the moon jellyfish *Aurelia* undergoes spawning, planula settle to produce a structure called a strobila, from which each segment that buds off becomes a juvenile medusa (Brekhman et al. 2015). In this brief comparison, it seems as though the medusae are more limited in their regenerative abilities. It is possible that this life stage is not capable of healing and regenerating in the traditional sense due to its more complex structure. The full extent of variation in life history and how it contributes to regeneration remains to be determined, but the diversity within Cnidaria makes it a unique group in which to investigate this potential link.

The extent of regeneration competency across cnidarians is not yet well characterized, but recent work suggests regeneration is possible in polyps, sea anemones, and to some extent even medusae. *Hydra* are well known and have long been studied as a model for regeneration (Galliot and Schmid 2002; Bode 2003; Galliot 2012). *Hydra* have an incredible regenerative capability, being able to regenerate head and foot when bisected and even capable of generating full polyps from cell aggregates (Trembley 1744; Gierer et al. 1972; Technau et al. 2000; Vogg et al. 2019). Similar to *Hydra, Nematostella* are another cnidarian species that have been widely studied and are also capable of full body regeneration (Layden et al. 2016). Recent research has expanded to investigate the tentacle and oral disc regeneration of sea anemones *Calliactis* and *Exaiptaisia* (Stewart et al. 2017; van der Burg et al. 2020). It is hypothesized that the regenerative abilities of sea anemones are due to their lack of a medusa stage and capacity to reproduce asexually (van der Burg and Prentis 2021). While medusae are seemingly more complex organisms, they may also possess the ability to regenerate. As an example, juvenile*Aurelia* use their muscular network to rearrange existing body parts after injury to achieve symmetry (Abrams et al. 2015). On the other hand,*Clytia* medusae are capable of healing and regenerating their umbrella and organs by tissue remodeling, cell proliferation, and cell migration (Sinigaglia et al. 2020). The immortal jellyfish *Turritopsis dohrnii*, when stressed or damaged, reverts from a medusa to a polyp stage through cell transdifferentiation and changes in expression of genes associated with aging, development, tissue differentiation, transposable elements (TEs), and undescribed functions (Matsumoto et al. 2019). Lastly, *Tripedalia* can regenerate a sensory structure called a rhopalium, which contains eyes, gravity sensors, and neurons (Stamatis et al. 2018). The extent of whole organisms or organ regeneration across the phylum remains an open question and a fruitful field for future investigation.

To advance our understanding of regeneration, it is imperative to expand investigations into comparative studies such as within Cnidaria. Comparisons of species within this phylum will reveal similarities and differences in the features and mechanisms used for regeneration. While the phylogenetic relationships within the speciose phylum are not fully resolved, major relationships are known, and the phylogenetic placement of widely studied species has also been identified (DeBiasse et al. 2024). Using a phylogenetic framework, researchers interested in regeneration can seek to address questions such as: (1) What are the cell types used for regeneration? (2) What are the cell mechanisms of regeneration? (Do cells dedifferentiate? Transdifferentiate? Is there an organizer cell that arises?) (3) What genetic pathways are used for healing and repatterning? and (4) What are the gene regulatory networks that underlie regeneration? (Fig. S1). These studies will provide fundamental knowledge as to the nuances of this process. By characterizing the genomic features that underlie life histories, development, and regeneration, we can identify components that are shared within the phylum. Subsequently, expanding these comparisons to Bilateria, we can determine features that are conserved across regenerating animals giving insight into the evolutionary history of regeneration.

## Determining functional genomics of regeneration in Cnidaria

The advancement of sequencing technologies achieved during the last decade allowed researchers to investigate genome-wide effects on traits of interest. Such studies have revealed additional genes involved in regeneration than were previously known. As an example, in *Hydra*, a fundamental study using *in situ* hybridization of candidate developmental genes uncovered a role of Wnt3 signaling in head regeneration (Hobmayer et al. 2000). Transcriptomic studies expanded on this work by characterizing the expression, timing, and regulation of pathways involved in animal response to injury and patterning (Petersen et al. 2015; Cazet et al. 2021; Murad et al. 2021). In addition to Wnt signaling, we now have a better understanding of the potential contributions of factors such as Jun, Fos, Fox, Otx, Creb, EGR, Rfx, Pax, and Zic (Petersen et al. 2015; Cazet et al. 2021; Murad et al. 2021). Similarly, a transcriptomic study in *Nematostella* characterized the expression of homeobox genes and transcription factors during oral and aboral regeneration and highlighted the overexpression of genes associated with developmental pathways (Schaffer et al. 2016). A comparison of genes involved in regeneration between planaria and *Nematostella* found *Otx* and *Six* involved in planaria oral and *Nematostella* aboral regeneration (Schaffer et al. 2016). Meanwhile, *SoxB, Wnt2*, and *FoxD* were associated with head regeneration in both (Schaffer et al. 2016). A similar pattern arises when we look at regeneration genes in *Hydra* and the acoel *Hofstenia*. In *Hofstenia, Wnt3, Brachyury, Sp5*, and *FoxA1* associated with *Hydra* head regeneration were found to function in posterior regeneration (Ramirez et al. 2020). These comprehensive studies provide a wide suite of genes and transcription factors to investigate to decipher the components and molecular mechanisms of regeneration. To determine the extent to which these patterns of gene expression are shared across organisms, we need to sample more broadly and use a comparative framework.

Comparative studies seek to identify conserved features of regeneration. Thus, a focus on known injury response and developmental pathways is intuitive. Yet, transcriptomic studies have demonstrated that, while similar pathways may be used, the processes may take different trajectories. As an example, a study comparing *Hydra* head regeneration and head development during budding found that gene expression was more dynamic during regeneration (Murad et al. 2021). This study found that the head organizer gene, *Wnt3*, increased in expression throughout budding, but during regeneration, *Wnt3* is lowly expressed early on and peaks around 12 h post-bisection and then decreases in expression again (Murad et al. 2021). Similarly, 298 genes had different expression profiles and different genetic trajectories during budding and regeneration (Murad et al. 2021). These results suggest regeneration does not recapitulate development, and that there may be more unexplored distinctions to this process. While a focus on known developmental genes has increased our understanding of regeneration, it has also limited our attention to a handful of pathways. The conservation of gene regulatory networks and underlying molecular processes of comparative regeneration remain to be determined (Srivastava 2021). Genome-wide approaches have discovered genes important to regeneration that are species-specific, uncharacterized, or novel (Petersen et al. 2015; Bryant et al. 2017; Stewart et al. 2017; Matsumoto et al. 2019). Future studies should begin to decipher the actions of these uncharacterized and novel genes, which may carry out unique functions during regeneration. Uncovering the functions of these novel and species-specific genes will help us better understand the mechanisms of regeneration that are currently limited due to a focus on identifying conserved genetic pathways for regeneration. Moreover, this understanding may help resolve the evolutionary history of regeneration.

## Using multi-omics approaches to shed light on regeneration

Multi-omics is the practice of combining multiple “omics” datasets to better understand a pathway or biological process. Some examples of these -omics include genomics (genome content), transcriptomics (gene expression), proteomics (protein), metabolomics (metabolites), and microbiomics (microbiome) (Hasin et al. 2017). The approach of combining these data types gives information about complex processes from multiple levels of biological organization. Multi-omics studies have the potential to bridge the knowledge gap between genotype and phenotype relationships. As an example, RNA-seq (transcriptomics) studies tell us which genes are expressed differentially between treatments or tissue types. Combining RNA-seq with ChIP-seq or ATAC-seq (chromatin accessibility) reveals areas of *cis*-regulation, transcription factor binding, and the regulatory effects on gene expression. This combination allows us to create putative gene regulatory networks and to visualize how pathways proceed and interact. An added level of validation comes from using proteomics or metabolomics, which can confirm mRNA translation, protein structure, protein–protein interactions, and the physiological outcome of metabolic processes. Although a promising field, some limitations of multi-omics approaches include issues with normalization, sampling bias, and poorly designed experiments (Krassowski et al. 2020). Future studies should consider statistical power and reproducibility in designing experiments that may reveal additional genes, proteins, or pathways that contribute to their process of interest.

In Cnidaria, previous studies combined RNA-seq, ATAC-seq, and ChIP-seq to identify developmental pathways important for regeneration (Cazet et al. 2021; Murad et al. 2021). With advances in single-cell genomics, we can now determine gene regulation at the single-cell level by combining scRNA-seq and scATAC-seq. Peterson et al. combined time series transcriptomics and proteomics to identify genes and proteins important for injury response and repatterning (Petersen et al. 2015). By combining the two data types, the list of candidate genes more than doubled, and novel genes and proteins associated with regeneration were identified (Petersen et al. 2015). One thing to keep in mind when combining data sets is the time frames relevant to linking genomics and proteomics studies. For example, in mammals, an increase in mRNA results in a change of protein concentration anytime between minutes to 2–3 days (Hargrove et al. 1991). Metabolomics can be used to complement the above approaches to identify metabolic pathways that are being upregulated or downregulated during healing and regeneration. For example, research has shown that stress and immune response are important for regeneration, and the metabolic pathways for these processes are known in some model organisms. Proteomic studies found increased activity of metabolic pathways during planaria and mice liver regeneration (see review Franco et al. 2013). Metabolomics can be used to investigate the activity of metabolic pathways during regeneration to determine whether any facilitate the process of healing or injury response. Metabolic studies during regeneration may confirm the function and timing of these pathways and identify new ones important for regeneration. Furthermore, a rising amount of research is being done to investigate the role of microorganisms on cnidarian immunity, development, and regeneration (Bosch et al. 2014; Parisi et al. 2020; Klein et al. 2021). An open question is whether host/microbe relationships may enhance cnidarian immune systems thus contributing to healing during regeneration. A recent study found that anti-microbial treated *Nematostella* and *Aiptasia* regenerated slower than control animals, and treatment resulted in fewer and smaller regenerated tentacles suggesting a role for microorganisms in cnidarian regenerative ability (Da-Anoy et al. 2025). This integration of microbiomics has the potential to decipher the role of external factors in regeneration competency. These studies have an important role in understanding regeneration and are also important in the context of species resilience and conservation.

## Identifying cell types of regenerating tissues

While some organisms have stem cells that can produce different cell types during regeneration, other regenerating organisms make use of multipotent or unipotent cells (Tanaka and Reddien 2011). The actions of organizing cells after injury, the constraints or malleability of cell identities, and the trajectories taken by differentiating cells during regeneration are understudied. We can now begin to address this gap in knowledge using single-cell sequencing and analyses. Advances in single-cell technologies now facilitate cell identification and lineage tracing. As an example, a study in *Xenopus* tadpoles identified a regeneration-organizing cell type that allowed for regeneration competence (Aztekin et al. 2019). These cells are found in the wound epidermis and express genes involved in regeneration signaling pathways such as FGF, Wnt, BMP, Msx, and Notch (Aztekin et al. 2019). On the other hand, single-cell analyses in *Axolotl* found that connective tissue cells dedifferentiate into a multipotent state and then redifferentiate during limb regeneration (Gerber et al. 2018). By tracking the movement and gene expression of connective tissue cells, researchers were able to detect a restriction in source cells recruited by different areas of the regenerating limb. Similar studies using single-cell technologies in other organisms can help identify cell types that may play a large role in regeneration. Determining the cell types that are used by different regenerating animals and the cell states or gene expression trajectories that they take will help identify potential homologies or convergence in the process.

Cnidarians are diploblastic animals, with two germ layers as opposed to the three found in bilaterian animals. Although seemingly simple in structure, they possess epithelial, muscle, gland, neuron, stem, and novel stinging cells (Sebé-Pedrós et al. 2018; Siebert et al. 2019). There is now an increasing amount of knowledge about the cell types and trajectories of some model cnidarians, including *Nematostella, Hydra, Clytia*, and *Hydractinia* (Sebé-Pedrós et al. 2018; Siebert et al. 2019; Chari et al. 2021; Salamanca-Díaz et al. 2025). These cell atlases were generated with adult organisms, but as technologies improve, there is a push to incorporate developmental time points and an opportunity to study regeneration dynamics at the single-cell level. This knowledge can be leveraged to investigate cell signaling, cell specification, and cell movement during regeneration. For example, *Hydra* have 3 stem cell lineages whose differentiation trajectories have been projected and described in detail (Siebert et al. 2019; Cazet et al. 2023). Yet, the movement of cells and whether the same differentiation trajectories are taken during regeneration and development remain to be discovered. *Hydra* also have a cluster of cells known as the head organizer that maintains axial patterning and controls head regeneration (Bode 2012). The cellular composition and signaling mechanisms of the head organizer remain elusive. Single-cell sequencing during a regeneration time course may help decipher the cellular substructure and signaling of the *Hydra* head organizer. This type of analysis will reveal similarities or differences between a “steady state” organizer and “regenerating” organizer. Similar studies can be done to compare the mechanisms of regeneration in other cnidarians where the presence and potency of stem cells are unknown (Gold and Jacobs 2013). Moreover, cell atlases and cell trajectory analyses from regenerating tissues can help identify potential organizing cells that arise after injury and may reveal whether dedifferentiation or transdifferentiation (mechanisms in regenerating vertebrate models) takes place in addition to the use of stem cells. By investigating the identity of cells deployed for regeneration and their molecular signaling, we can detect the elements that underlie malleability and constraints of cells to differentiate. For example, if organizer cells are detected in regenerating cnidarians, their gene expression profiles can be compared to those of the *Xenopus* cell organizer to determine whether there are any unifying principles. Similarly, if new cell states arise in cnidarians due to dedifferentiation, similar to axolotls, we can probe the characteristics of these cell types that make them primed to undergo this transition. This knowledge can be applied to engineer cells and manipulate regeneration competency in other animals.

## Gene editing to validate candidate features of regeneration competency

Previous and future genetics studies will identify genomic components important for regeneration. Historically, gene knockdowns or chemical inhibitions allowed researchers to validate the association of some key pathways in regeneration. However, this was limited to a few genes and a few model organisms. The advent of CRISPR technology now allows researchers to identify and validate specific gene functions in many non-model organisms. For regeneration studies, CRISPR knock-ins can be used to apply fluorescent tags to genes that are cell-specific, injury-related, or candidate regeneration genes to visualize their expression. In addition, knockout animals can be generated and tested for regenerative ability. A potential limitation is that knocking down development associated genes may be detrimental to an organism. If organisms are malleable to knock-in approaches, this can be used to generate an inducible system to target developmental genes during regeneration (Cao et al. 2016). Alternatively, knockdown approaches using electroporation of shRNA and RNAi have been established in cnidarians (Karabulut et al. 2019; Quiroga-Artigas et al. 2020).

Cnidarians are a good system for gene editing protocols to uncover the genetic underpinnings of regeneration. Firstly, some cnidarians are small and easy to maintain in a lab. A few species are being established as model organisms for studies in stem cell biology, neurogenesis, aging, development, and regeneration. Secondly, many of them are capable of asexual reproduction, which allows for both large populations and genetically identical individuals. Currently, standardized protocols for care and husbandry have been published for *Nematostella* and *Clytia* (Lechable et al. 2020; Carvalho et al. 2023). Gene editing methods are also already in place for *Nematostella, Hydractinia*, and to some extent *Hydra* (Lommel et al. 2017; Nakanishi and Martindale 2018; Sanders et al. 2018; Carvalho et al. 2023). These standardized protocols can be used as a starting point to investigate regeneration in some of these lab reared cnidarians and then expanded to additional species. As we know, there are potentially different mechanisms, cell types, and competency of regeneration for cnidarians, comparative gene editing studies will reveal the exact function of genetic components and whether the same genes and gene networks are used for regeneration within the phylum.

## Additional considerations

Genomic components that have been historically overlooked in regeneration due to limited genomic resources are TEs. Long thought of as “junk” DNA, recent studies have identified a potential role for TEs in mammal development (Low et al. 2021; Senft and Macfarlan 2021). In regenerating animals, TEs were dynamically expressed during regeneration in the sea cucumber *Holothuria glaberrima*, in *Hydra*, and in *Turritopsis* (Mashanov et al. 2012; Petersen et al. 2015; Matsumoto et al. 2019). In addition, it was recently hypothesized that regulation of TEs is important for regeneration competency across animals (Angileri et al. 2022). In *Hydra*, “active TEs” were found to be responsible for changes in genome length between two strains, and some active TEs were found to be inserted and expressed in stem cells (Kon-Nanjo et al. 2024). The specific actions of TEs during regeneration remain unknown. Specifically, if any families are under selection or whether TEs have a direct role in gene regulation during regeneration is an open question. Determining whether TEs have a functional role in regeneration is a tall task because TEs remain difficult to classify and study. Future research should aim to characterize TEs in cnidarian genomes, investigate their expression profiles (transcriptomics or *in situ* if possible), and attempt to decipher their specific functions with gene silencing approaches.

## Conclusions

We are now entering an exciting new chapter for comparative genomics studies. Advances in sequencing technologies and bioengineering make it possible to expand out from model organisms into diverse species and traits. Cnidarians are a unique system for comparative studies on regeneration due to their phylogenetic placement, diverse life histories, and regenerative capacity. Cnidarians represent a growing field of interest with many open questions for investigation. Genome-wide approaches have identified components of the genome that have until now been under investigated and may have an important role in regeneration. Combination of multi-omics approaches can now reveal the missing links between genotype and phenotype relationships in regeneration. Single-cell genomics allow us to identify cell types and trace their differentiation trajectories. Lastly, transgenics in multiple organisms can be used to validate gene functions. A comparative approach of regenerating animals with different regeneration potentials will help decipher the mechanisms, cellular, and genetic components of regeneration. As we deepen our understanding of the mechanisms underlying regeneration, it is now crucial to elucidate its evolutionary history. Investigating the factors that underlie regeneration across animals will unravel homologies or instances of co-option. To do this, we must pivot away from a focus on a few model organisms to investigating regenerations in a broad range of taxa, including early branching organisms. Understanding the shared components of regenerating organisms, or unique adaptations, holds the potential to manipulate regenerative ability using gene therapy and bioengineering.

## Supplementary Material

icaf006_Supplemental_File

## Notes

From the symposium “Cnidarian sensory systems as comparative models for the evolution of complexity” presented at the annual meeting of the Society for Integrative and Comparative Biology, January 3-7th, 2025.
