# Supplementary material for: Investigating the evolution and features of regeneration using cnidarians
Source: Integr Comp Biol. 2025 Mar 17;65(3):713–26. doi: 10.1093/icb/icaf006 (PMC12464823; doi:10.1093/icb/icaf006)
Supplement: icaf006_Supplemental_File [file icaf006_supplemental_file.pdf]

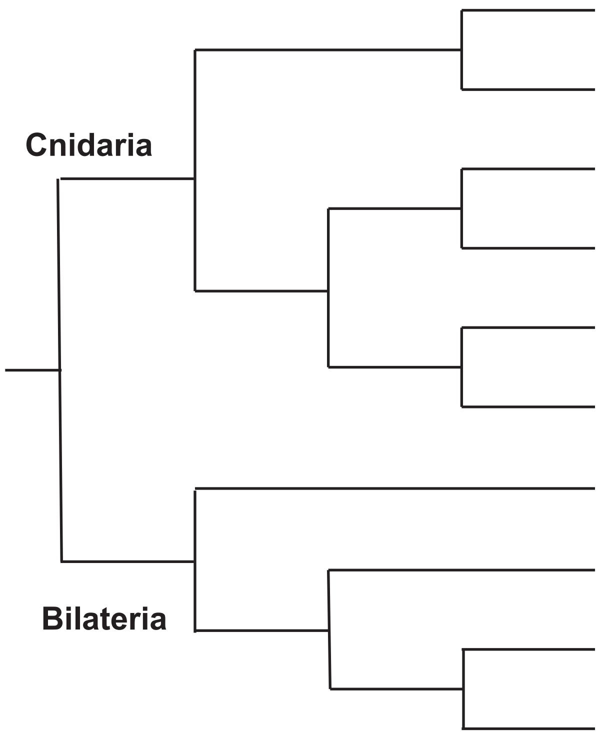

	Adult whole-body regeneration?	piwi+ cells?	stem cells (how many)?	GRN
Nematostella	Yes	Yes (pleuripotent)	Yes, pSC (2?)	
Exaiptasia	Yes	Yes (pleuripotent)	TBD	
Tripedalia	No	TBD	TBD	
Aurelia	No?	TBD	Yes, interstitial	
Hydra	Yes	Yes (pleuripotent)	Yes, 3	MAPK? → Wnt; bZIP → Wnt
Clytia	No?	Yes (pleuripotent)	Yes, 2?	
Hofstenia	Yes	Yes (pleuripotent)	Yes, neoblast	EGR → Wnt, Follistatin, Runt, Neuregulin
Schmidtea	Yes	Yes (pleuripotent)	Yes, neoblast	MAPK? → EGR, Wnt, Follistatin, Runt
Axolotl	No	Yes (lineage-restricted)	multipotent cells	
Xenopus	No	Yes (lineage-restricted)	multipotent cells	

Additional considerations: 1) Do organizing cells arise during regeneration? 2) Do cells dedifferentiate or transdifferentiate during regeneration?

Figure S1. Comparative framework to investigate regeneration. Adapated from Srivastava 2021. Our knowledge of cnidarian regeneration mostly comes from *Hydra*, a long established species. Yet, recent studies and a growth in the cnidaria field of research highlights that there may be diversity to unearth. This may give insight to the evolutionary history of regeneration.
